# Sexual Health Determinants of Normal Weight, Overweight, and Obese Sexual Minority Men

**DOI:** 10.1155/2021/1272316

**Published:** 2021-03-18

**Authors:** Henrique Pereira

**Affiliations:** ^1^Department of Psychology and Education, University of Beira Interior, Covilhã 6200-209, Portugal; ^2^The Health Sciences Research Centre (CICS-UBI), Covilhã, Portugal; ^3^The Research Center in Sports Sciences, Health Sciences and Human Development (CIDESD), Vila Real, Portugal

## Abstract

**Background:**

With the growing recognition of overweight and obesity as significant, international public health concerns, the body of research investigating the relationship between body mass index (BMI), sexual health, and sexual functioning in sexual minority men is still scarce.

**Objective:**

The purpose of this study is to assess sexual health determinants (sexual behavior and sexual functioning) in relation to normal weight, overweight, and obesity among gay and bisexual men.

**Methods and Materials:**

The survey included four categories of questions/measurements, encompassing sociodemographic information, protected/unprotected sexual behaviors, sexual functioning, and BMI. The survey was conducted online, and recruitment consisted of online notifications (emails and electronic messages) and advertisements sent to LGBT community organizations, mailing lists, and social networks.

**Results:**

The study sample was composed of 741 gay and bisexual men, ranging in age from 21 to 75 years (*M*_age_ = 43.30, SD_age_ = 11.37); 62.5% of men self-identified as gay and 37.5% as bisexual. Prevalence of normal weight was 50.3%, of overweight, 33.3%, and of obesity, 16.4%. Participants with overweight and obesity showed a lower frequency of anal receptive sex without condoms when scompared to participants with normal weight. Hierarchical multiple regression analysis to assess the effects of BMI on sexual health showed that being younger in age, self-identifying as gay, being in a relationship, having longer penises, adopting insertive position in sex, and being normal weight were significant predictors of anal receptive sex without condoms, explaining 24.2% of the total variance. Yet, BMI was not predictive of sexual functioning.

**Conclusion:**

These findings highlight the importance of including BMI in sexual behavior models of sexual minority men to better understand BMI's role in influencing sexual risk.

## 1. Introduction

Overweight and obesity are defined as abnormal or excessive fat accumulation that may impair health and considered by the World Health Organization [1] as one of the most concerning preventable health problems in the Western World. However, it does not affect all population groups equally and sexual minority individuals have been identified as a high-risk group, due to exposure to specific stressors pertaining to a sexual minority [2, 3], namely, homophobia, fear of rejection, and concealment, leading to increased levels of anxiety and the need to find coping mechanisms to mediate stress, including overeating [4].

Self-identified gay and bisexual men (GBM) seem to be particularly vulnerable to weight stigma, sexual objectification, and social comparison [5], leading them to show a lower prevalence of overweight and obesity compared to straight men [6–9] although some research has not found any significant differences [10]. Nevertheless, recent studies have contributed to this clarification, demonstrating that GBM are characterized by increased probability of being overweight and obese [11, 12].

The psychosocial consequences of overweight and obesity, including depression, social difficulties, and low sexual frequency, may negatively influence the ability to have and maintain well-balanced relationships, and safer sex practices become more difficult to negotiate, thus leading to increased sexual risk [13]. Moreover, self-identified gay and bisexual men with overweight and obesity may be less likely to engage in safer sexual practices because they may lack self-esteem and/or self-efficacy due to fear of rejection about their weight [14].

Several studies have been conducted among GBM to explore health determinants of overweight and obesity, such as education attainment (high education attainment was protective against obesity status) [15]; perceived weight status, body dissatisfaction, and self-objectification (participants with obesity scored significantly higher on measures of body dissatisfaction and lower on measures of sexual sensation seeking) [16]; binge eating behavior, or any disordered eating behavior (GBM showed more prevalence of binge eating and eating behavior disorders) [17]; lower self-esteem [18]; or higher LGB climate scores (more supportive environments were associated with lower risk of overweight and obesity) [19].

While there have been attempts to explore the association of obesity and risky sexual behaviors among GBM, findings have been contradictory. For instance, Guadamuz et al. [20] found no significant association between obesity and sexual risk-taking behaviors but found high prevalence of overweight and obesity in this population, whereas Allensworth-Davies et al. [21] found that men with overweight and obesity engaged in less unprotected anal intercourse. However, Moskowitz and Seal [22] found that increased body mass index (BMI) was associated with decreased condom use. Similarly, Kraft et al. [23] found that GBM with overweight were 3.6 times more likely than GBM with obesity to have had unsafe sex. Motivational factors may best explain BMI-related sexual experience whereby less confident individuals harbor negative expectations about dating and are then less likely to initiate or respond to sexual opportunities [24].

Overweight and obesity may have profound medical, psychological, and emotional consequences and are associated with sexual difficulties [25]. Yet, very little is known regarding the interrelationship between obesity and sexual functioning in GBM. Some studies bring evidence to the fact that the prevalence of erectile function is significantly higher in overweight men and in men with obesity in general [26], and in GBM in particular [27].

After more than four decades of homophobic dictatorship in Portugal and the importance of the Catholic Church, the country now lives in a protective and inclusive sociopolitical environment for sexual minorities, exhibited through the existence of a non-discrimination clause based on sexual orientation in the Portuguese constitution, the 2010 law authorizing same-sex marriage, and the 2016 law allowing adoption by same-sex couples [28, 29]. However, the identity experience of LGB people is still conditioned by the existence of negative attitudes, prejudice, and sexual stigma [30], which, through the effects of minority stress, may have a negative impact on physical and mental health [31].

With the growing recognition of overweight and obesity as significant, international public health concerns, the body of research investigating the relationship between obesity, sexual health, and sexual functioning in GBM is still scarce. Given the high prevalence of HIV and other sexually transmitted diseases in gay and bisexual men [32], its associated mental health concerns [33], and subsequent possible associations with BMI in Portuguese social environment (where sexual stigma still exists), this study was carried out to address a gap in the literature, since there is a lack of available studies in this field. Therefore, the purpose of this study is to assess sexual health determinants (sexual behavior and sexual functioning) in Portuguese GBM with normal weight, overweight, and obesity.

## 2. Materials and Methods

The survey included four categories of questions/measurements, encompassing sociodemographic information, protected/unprotected sexual behaviors, sexual functioning, and BMI.

### 2.1. Sociodemographic Information

Items included age, gender, sexual orientation (using the self-report label of “gay” or “bisexual”), marital status, place of residence, educational attainment, professional status, HIV status, number of times they had sex per week, level of acceptance of sexual identity, self-assessment of penis length, and satisfaction with penis length.

### 2.2. Sexual Behaviors

Participants were asked to recall their sexual behavior during the previous 6 months. The study collected information regarding the frequency of receptive and insertive anal sex, as well as condom use during sexual intercourse.

### 2.3. Sexual Functioning

The Portuguese version of the Massachusetts General Hospital-Sexual Function Questionnaire (MGH-SFQ) [34] was used. This is a 5-item questionnaire that assesses sexual interest, arousal, orgasm, erection, and general sexual satisfaction on a scale 1–7 (totally absent—completely above normal). An excellent Cronbach's alpha of .91 was obtained for reliability analysis.

### 2.4. BMI

Body mass index was calculated from respondents' self-reported weight and height (BMI = [weight (kg)]/[height (m)]^2^) and then coded into three categories. BMI categories were defined as normal weight (18.5 kg/m^2^ ≤ BMI b 25 kg/m^2^), overweight (25 kg/m^2^ ≤ BMI b 30 kg/m^2^), and obese (BMI ≥ 30 kg/m^2^).

The survey was conducted online between January 2020 and February 2020. Recruitment consisted of online notifications (emails and electronic messages) and advertisements sent to LGBT community organizations, mailing lists, and social networks, such as Facebook. Participants responded voluntarily to the study's outreach online through a website created for this purpose, and no compensation was provided. All advertisements referred participants directly to the online survey, where they were informed that their responses would be anonymous and confidential, in accordance with the Helsinki Declaration of ethical principles concerning research involving human subjects. The first page of the questionnaire explained the study's objectives and informed participants about how to complete the survey, their freedom to withdraw from the study at any time, and how to contact the author for further information about the study, if needed. Confidentiality was ensured by using codes on documents containing study data, by encrypting identifiable data, by assigning security codes to computerized records, and by limiting access to identifying information (e.g., IP addresses).

## 3. Results

### 3.1. Sociodemographic Information

The study sample was composed of 741 GBM, ranging in age from 21 to 75 years (*M*_age_ = 43.30, SD_age_ = 11.37); 62.5% of men self-identified as gay and 37.5% as bisexual. The majority of the overall sample was single (48.4%), held a university degree, and lived in large urban environments. 63.4% were employed and the vast majority (80.1%) said that they were HIV-negative. On average, participants reported having sex 1.76 (SD = 1.45) times per week, and their level of acceptance of their gay or bisexual identity was relatively high (8.42 on a scale from 1 to 10). Finally, self-assessment of penis length and levels of satisfaction with penis length were also measured (*M* = 16.91 cm for penis length; 7.73 for levels of satisfaction with penis length, on a scale of 1 to 10). Prevalence of normal weight was 50.3%, of overweight 33.3%, and of obesity 16.4%. No significant differences were found for BMI across age groups. Study participants' sociodemographic information is described in greater detail in Table 1.

### 3.2. BMI, Sociodemographic Characteristics, Sexual Practices, and Sexual Functioning

Table 2 shows the results obtained for multiple variables by BMI categories. Regarding sociodemographic characteristics, significant differences were found for “educational attainment” [*X*^2^(2) = 8.051; *p* = 0.018], indicating that more participants with overweight and obesity had attained university degrees. Regarding sexual practices, significant results were found for “frequency of receptive anal sex without condoms” [*F*(2; 245) = 3.872; *p* = 0.022], indicating that participants with overweight and obesity showed lower frequency of this sexual practice when compared to participants with normal weight. No significant differences were found for any parameter of sexual practices, despite the fact that participants with overweight and obesity showed lower scores of general sexual functioning. A significant difference was found when comparing self-assessment of penis length by BMI categories [*F*(2; 251) = 6.023; *p* = 0.003]: participants with overweight and obesity said they had shorter penises when compared to men with normal weight.

Finally, two hierarchical multiple regression analyses were performed to assess the effects of BMI on sexual health (anal receptive sex without condoms and general sexual functioning). The first model (BMI predicting anal receptive sex without condoms) was chosen because this is considered the riskiest sexual practice for HIV transmission and other sexually transmitted diseases. The possible confounding variables “age,” “sexual orientation,” and “marital status” were added in the first block. Levels of acceptance of sexual identity, penis length, satisfaction with penis length, HIV status, and sexual role were added in the second block. BMI was added in the third block. The first block of the analysis explained 15.9% of the overall variance, the second block explained 22.7%, and the third (where BMI was included) explained 24.2%. Therefore, as shown in Table 3, being younger in age, self-identifying as gay, being in a relationship, having longer penises, adopting insertive position in sex, and being normal weight were significant predictors of anal receptive sex without condoms. The second model (BMI predicting sexual functioning) was chosen after a single variable encompassing all five items of the MGH-SFQ was created. The first block of the analysis explained 0.29% of the overall variance, the second block explained 14.3%, and the third (where BMI was included) explained 14.7%. Therefore, as shown in Table 4, only being in a relationship and having higher levels of acceptance of sexual identity (and not BMI) were significant predictors of higher levels of sexual functioning. Effect size was medium for the anal receptive sex without condoms model, and small for the sexual functioning model.

## 4. Discussion

Using a convenience sample of 741 Portuguese sexual minority men, this study explored sexual health characteristics of self-identified gay and bisexual adult men with normal weight, overweight, and obesity. Some previous research has found that sexual minority populations may be disproportionally impacted by overweight and obesity [35], and this remains a significant public health issue, so related outcomes, such as sexual health characteristics, should be studied among sexual minority men in order to contribute to a better understanding of these complex associations.

Consistently with other studies [20], a high prevalence of being either overweight or obese was found (around 50%), specifically, 33.3% overweight and 16.4% obese, and contrary to other studies [7, 8], greater prevalence of healthy weight was not found. With respect to BMI correlates, significant differences were only found for education, frequency of receptive anal sex, and penis length. Regarding education, results are not consistent with other studies [15] that demonstrate the protective effect of education attainment against obesity. Overeating, for instance, may be utilized as a coping mechanism to deal with stress that comes from exposure to an adverse social heterosexist environment due to homophobia and biphobia [36], but, on the other hand, more educated men may have more resources to feel less pressured to engage in social comparison actions that are common in gay and bisexual communities that are oversexualized because of weight stigma, sexual objectification, and body image [5, 37]. Regarding penis length and BMI, results were consistent with other studies that state that men with obesity have significantly shorter and clinically buried penises [38, 39]. Since penis length is usually measured against the pubic symphysis, for men with overweight and obesity that accumulate more fat at the base of the penis, the less of the said penis becomes visible to the naked eye, even if in self-reports of penis size many men tend to over-report [38]. Regarding the frequency of receptive anal sex without condoms, results were similar to those obtained by other researchers [20, 21, 23] and did not replicate other findings that sexual minority men with obesity engage in more risky sexual activities [22]. These results suggest that BMI could be an important factor to consider when designing HIV/STD prevention programs since men without obesity appear to be at greater risk for HIV/AIDS and other STDs due to their and their partners' increased probability to engage in unprotected sex [40].

Being younger, self-identifying as gay, being in a relationship, having longer penises, adopting insertive position in sex, and being normal weight were significant predictors of anal receptive sex without condoms. The positive relationship between these characteristics and unsafe sex may be explained because such men may have a sense of invulnerability to disease due to feelings of attractiveness and confidence from being younger, virile, and non-obese, or because they are more sexually active overall, thus leading to a higher probability of having unsafe sex [41], or even because sexual partners of these men are less likely to insist on condom use, because they believe that they are less likely to be infected with an STD or because they are reluctant to insist on safer sex for fear of losing their sexual opportunity with these attractive men [23].

However, being in a relationship and having higher levels of acceptance of sexual identity were the only significant predictors of higher levels of sexual functioning. Unlike other studies that found that overweight and men with obesity were more likely to present sexual dysfunctions, these positive protective factors may be directly contributing to better levels of mental health, thus helping maintain a normal sexual functioning [19, 32]. Consequently, an association of sexual problems with obesity was not found; perhaps, this has to do with the fact that there is strong evidence of the relationship between sexual functioning and testosterone in men [42] whereas in overweight and obesity, sexual dysfunctions seem to be more attributable to morbid obesity [43].

Sexual orientation, penis length, and sexual role significantly predicted anal receptive sex without condoms, but not sexual functioning. This may have to do with the fact that sexual functioning is a complex dimension of human sexual expression, with physiological and emotional components, namely, the acceptance of sexual identity [22, 44], whereas in the adoption of unprotected anal sex, BMI, penis size (also because of the thick pad of fat that envelops the shaft of the penis), and the type of role in sexual activity may compensate the sense of loss of masculinity and power associated with obesity [45].

This study is an important first look at the sexual health determinants of sexual minority men in relation to BMI categories in Portugal. BMI issues are inherently connected to social normative concepts of sexual identity and sexual desire and there is limited research that explores this in a Portuguese context. Sexual minority men and BMI characteristics are a public health concern [46] and interventions addressing unhealthy weight gain in sexual minority men must be relevant. Further studies to explore the association of sexual orientation and BMI may be that sexual minority groups are exposed to psychosocial stressors, which may influence their health behaviors such as diet or physical activity, or alcohol consumption indicated in weight gain and linked to increased risk of chronic health conditions such as diabetes and cardiovascular disease.

Several limitations ultimately restrict the ability to generalize the research findings. The study sample was disproportionately comprised of urban, well-educated GBM who possessed Internet and technological access and who were recruited through social organizations and social networks in Portugal. Consequently, the extent to which these men are representative of all gay and bisexual remains uncertain. It is possible that the surveyed sample consisted of a group that exhibited high acceptance of their sexual identity, compared to GBM who may not attend community organizations due to internalized homonegativity. In order to address this limitation, future studies should include non-community samples. Given that height and weight were self-reported by participants and that approximately 13% of men tend to misclassify themselves into a lower self-reported BMI category, especially in men with obesity [47], this is also a limitation. Future studies should include measurements. Also, this study only assessed those men who self-identify as gay or bisexual, leaving out those men who have sex with men and engage in anal receptive sex without condoms, but do not self-identify as gay or bisexual. Future studies should include these men.

However, the intention of this study was not to generalize its findings, but, rather, to contribute to a better understanding of the associations between sexual health determinants and BMI in a Portuguese sample of sexual minority men who live in a society that legally protects them, but is also a source of stigmatization. Furthermore, the fact that the study obtained its measurements from participant self-assessments, particularly weight, height, and penis size measurements, may create some concerns about measurement validity. These limitations aside, this study expands on the existing knowledge regarding sexual health and sexual functioning in relation to BMI among self-identified gays and bisexuals.

## 5. Conclusions

Weight status predicted engaging in anal receptive sex without condoms. These findings highlight the importance of including BMI in sexual behavior models of sexual minority men to better understand BMI's role in influencing sexual risk and sexually transmitted infections and HIV transmission. Advance in knowledge about the behavioral risk factors, including BMI, for having unsafe sex could potentially contribute to more effective approaches to reducing high risk among sexual minority men.

## Figures and Tables

**Table 1:** Sociodemographic characteristics of the sample participants (*n* = 741).

		*n*	*%*	*M*	SD
Age				43.30	11.37

Place of residence	Large urban environment	449	60.6		
	Small urban environment	214	28.9		
	Large rural environment	46	6.1		
	Small rural environment	32	4.3		

Marital status^a^	Single	359	48.4		
	Married to a man	19	2.5		
	Married to a woman	102	13.7		
	De facto union with a man	10	1.4		
	De facto union with a woman	53	7.2		
	Dating a man	62	8.3		
	Dating a woman	22	2.9		
	Divorced	114	15.6		

Educational attainment	Up to 12 years of schooling	265	35.8		
	University degree	476	64.2		

Occupation	Student	19	2.5		
	Unemployed	40	5.4		
	Working-student	10	1.4		
	Employed	470	63.4		
	Self-employed	150	20.3		
	Retired	13	1.8		
	Other	39	5.1		

Self-assessed HIV status	HIV-negative	594	80.1		
	HIV-positive	63	8.5		
	Does not know	63	8.5		
	Rather not say	21	2.9		
Number of times of sexual activity/week				1.76	1.45
Level of acceptance of sexual identity (1–10)				8.42	1.82
Self-assessment of penis length (cm)				16.90	2.00
Level of satisfaction with penis length (1–10)				7.73	2.14

BMI categories^b^	Normal weight	373	50.3		
	Overweight	247	33.3		
	Obese	121	16.4		

^a^In Portugal, same-sex de facto union is legal since 2001, and same-sex marriage since 2010. ^b^BMI categories are defined as normal weight (18.5 kg/m^2^ ≤ BMI b 25 kg/m^2^), overweight (25 kg/m^2^ ≤ BMI b 30 kg/m^2^), and obese (BMI ≥ 30 kg/m^2^).

**Table 2:** Results for multiple variables by BMI categories.

	BMI Categories (%/*M*/SD)			Chi^2^(df)/*T*(df)	*p*
	Normal weight	Overweight	Obese		
Sexual orientation					
Gay	33.9%	19.4%	9.7%	1.579(2)	0.454
Bisexual	16.9%	13.7%	6.5%		

Age					
21–37	18.4%	8.0%	4.0%	4.210(2)	0.378
38–47	16.8%	13.6%	6.4%		
48–75	15.6%	11.6%	5.6%		

Marital status					
Single	24.3%	16.9%	7.5%	15.986(14)	0.314
Married to a man	1.2%	0.8%	0.8%		
Married to a woman	7.1%	4.3%	3.1%		
De facto union with a man	0.4%	1.2%	0.0%		
De facto union with a woman	3.5%	2.7%	0.4%		
Dating a man	3.9%	2.4%	2.0%		
Dating a woman	0.4%	2.0%	0.4%		
Divorced	9.8%	3.1%	2.0%		

Educational attainment					
Up to 12 years of schooling	13.5%	14.3%	7.1%	8.051(2)	0.018 ^*∗*^
University degree	37.3%	19.0%	8.7%		

Place of residence					
Large urban environment	32.5%	20.8%	8.2%	4.065(6)	0.668
Small urban environment	13.3%	8.6%	5.9%		
Large rural environment	2.7%	2.0%	1.6%		
Small rural environment	2.0%	2.0%	0.4%		

Professional Status					
Student	0.8%	0.8%	0.4%	16.370(12)	0.175
Unemployed	3.5%	1.2%	0.8%		
Working-student	0.8%	0.8%	0.0%		
Employed	33.5%	17.7%	12.6%		
Self-employed	9.8%	9.4%	1.6%		
Retired	1.2%	0.8%	0.0%		
Other	0.8%	2.8%	0.8%		

Self-assessed HIV status					
HIV-negative	38.8%	28.8%	13.2%	4.209(6)	0.673
HIV-positive	6.0%	2.0%	0.8%		
Does not know	4.4%	2.0%	1.6%		
Rather not say	1.2%	0.8%	0.4%		

Frequency of receptive anal sex					
With condoms	2.65(1.38)	2.57(1.44)	2.37(1.29)	.664(2; 247)	0.516
Without condoms	2.00(1.11)	1.68(.95)	1.56(.95)	3.872(2; 245)	0.022 ^*∗*^

Frequency of insertive anal sex					
With condoms	2.68(1.41)	2.70(1.51)	2.35(1.32)	0.930(2; 244)	0.396
Without condoms	1.98(1.18)	1.98(1.22)	1.88(1.12)	0.118(2; 246)	0.889
Level of sexual identity acceptance (1–10)	8.40(1.90)	8.46(1.87)	8.36(1.69)	.042(2; 248)	.959
Self-assessment of penis length	17.24(1.73)	16.80(2.24)	16.05(1.825)	6.023(2; 251)	0.003 ^*∗*^
Satisfaction with penis length (1–10)	7.78(1.99)	7.93(2.31)	7.38(2.28)	0.905(2; 250)	0.406
Sexual interest	4.54(1.50)	4.21(1.66)	4.34(1.59)	1.171(2; 252)	0.312
Sexual arousal	4.60(1.35)	4.58(1.20)	4.48(1.47)	0.135(2; 248)	0.874
Orgasms	4.87(1.28)	4.72(1.35)	4.48(1.57)	1.358(2; 250)	0.259
Erectile function	4.84(1.39)	4.71(1.35)	4.48(1.51)	1.043(2; 250)	0.354
Satisfaction with sex life	4.60(1.38)	4.53(1.39)	4.50(1.41)	0.107(2; 249)	0.898
General Sexual functioning	4.69(1.15)	4.55(1.09)	4.38(1.36)	1.179(2; 252)	0.309

^*∗*^<0.05;  ^*∗∗*^<0.001.

**Table 3:** Hierarchical multiple regression analysis predicting anal receptive sex without condoms.

		*ß*	*t*	*p*	*R* ^*2*^	*F*	*p*
Model 1	Age	−0.209	−3.373	0.001 ^*∗*^	0.159	14.346	0.000 ^*∗∗*^
	Sexual orientation	−0.254	−4.121	0.000 ^*∗∗*^			
	Marital status	0.239	3.889	0.000 ^*∗∗*^			

Model 2	Age	−0.153	−2.459	0.015 ^*∗*^	0.227	8.147	0.000 ^*∗∗*^
	Sexual orientation	−0.269	−4.377	0.000 ^*∗∗*^			
	Marital status	0.229	3.811	0.000 ^*∗∗*^			
	Levels of acceptance of sexual identity	−0.046	−0.710	0.479			
	Penis length	0.238	3.364	0.001 ^*∗*^			
	Satisfaction with penis length	−0.089	−1.202	0.231			
	HIV status	−0.043	−0.710	0.478			
	Sexual role	0.182	3.035	0.003 ^*∗*^			

Model 3	Age	−0.135	−2.163	0.032 ^*∗*^	0.242	7.824	0.000 ^*∗∗*^
	Sexual orientation	−0.260	−4.258	0.000 ^*∗∗*^			
	Marital status	0.237	3.972	0.000 ^*∗∗*^			
	Levels of acceptance of sexual identity	−0.041	−0.639	0.523			
	Penis length	0.211	2.946	0.004 ^*∗*^			
	Satisfaction with penis length	−0.084	−1.132	0.259			
	HIV status	−0.049	−0.819	0.414			
	Sexual role	0.185	3.102	0.002 ^*∗*^			
	BMI	−0.127	−2.069	0.040 ^*∗*^			

^*∗*^<0.05;  ^*∗∗*^<0.001.

**Table 4:** Hierarchical multiple regression analysis predicting sexual functioning.

		*β*	*t*	*p*	*R* ^*2*^	*F*	*p*
Model 1	Age	−0.071	−1.088	0.278	0.029	2.369	0.071
	Sexual orientation	0.054	0.825	0.410			
	Marital status	0.156	2.406	0.017 ^*∗*^			

Model 2	Age	−0.074	−1.144	0.254	0.143	4.781	0.000 ^*∗∗*^
	Sexual orientation	0.116	1.834	0.068			
	Marital status	0.137	2.205	0.028 ^*∗*^			
	Levels of acceptance of sexual identity	0.291	4.376	0.000 ^*∗∗*^			
	Penis length	0.096	1.308	0.192			
	Satisfaction with penis length	0.033	0.420	0.675			
	HIV status	−0.029	−0.462	0.644			
	Sexual role	−0.034	−0.540	0.590			

Model 3	Age	−0.065	−1.004	0.316	0.147	4.355	0.000 ^*∗∗*^
	Sexual orientation	0.121	1.898	0.059			
	Marital status	0.141	2.262	0.025 ^*∗*^			
	Levels of acceptance of sexual identity	0.293	4.397	0.000 ^*∗∗*^			
	Penis length	0.083	1.108	0.269			
	Satisfaction with penis length	0.036	0.460	0.646			
	HIV status	−0.032	−0.513	0.608			
	Sexual role	−0.033	−0.523	0.601			
	BMI	−0.062	−0.976	0.330			

^*∗*^<0.05;  ^*∗∗*^<0.001

## Data Availability

The dataset used to support the findings of this study is available within the article.
